# Synthetic Lethality‐Based Targets and Their Exploration in Tumour Combination Strategies

**DOI:** 10.1111/jcmm.70756

**Published:** 2025-08-27

**Authors:** Lingya Wu, Yixuan Deng, Zhe Lei, Yuhong Wang, Shan Huang

**Affiliations:** ^1^ Department of Pathology The First Affiliated Hospital of Soochow University Suzhou Jiangsu China

**Keywords:** cancer therapy, combined strategy, DNA repair, PARP inhibitors, synthetic lethality

## Abstract

Synthetic lethality (SL) not only addresses the challenge of drug resistance associated with classical targeted therapies but also offers innovative therapeutic approaches for previously ‘undruggable’ targets, such as deletion mutations in tumour suppressor genes. Advances in technology have significantly enhanced our understanding of gene–gene interactions in cancer cells, enabling the identification of synthetic lethal targets and the development of drugs targeting these mechanisms. Following the extensive clinical application of PARP inhibitors—the first synthetic lethal targeted drugs approved for clinical use—emerging targets such as ATR, WEE1 and WRN have demonstrated promising clinical potential. This review examines the functions and molecular mechanisms underlying these targets and discusses recent advancements in the theory of synthetic lethality. Additionally, it emphasises the integration of synthetic lethal drugs with traditional cancer treatments, highlighting the clinical benefits of this combined strategy and its potential to facilitate more precise and individualised cancer treatment modalities in the future.

## 1 Introduction

**FIGURE 1:**
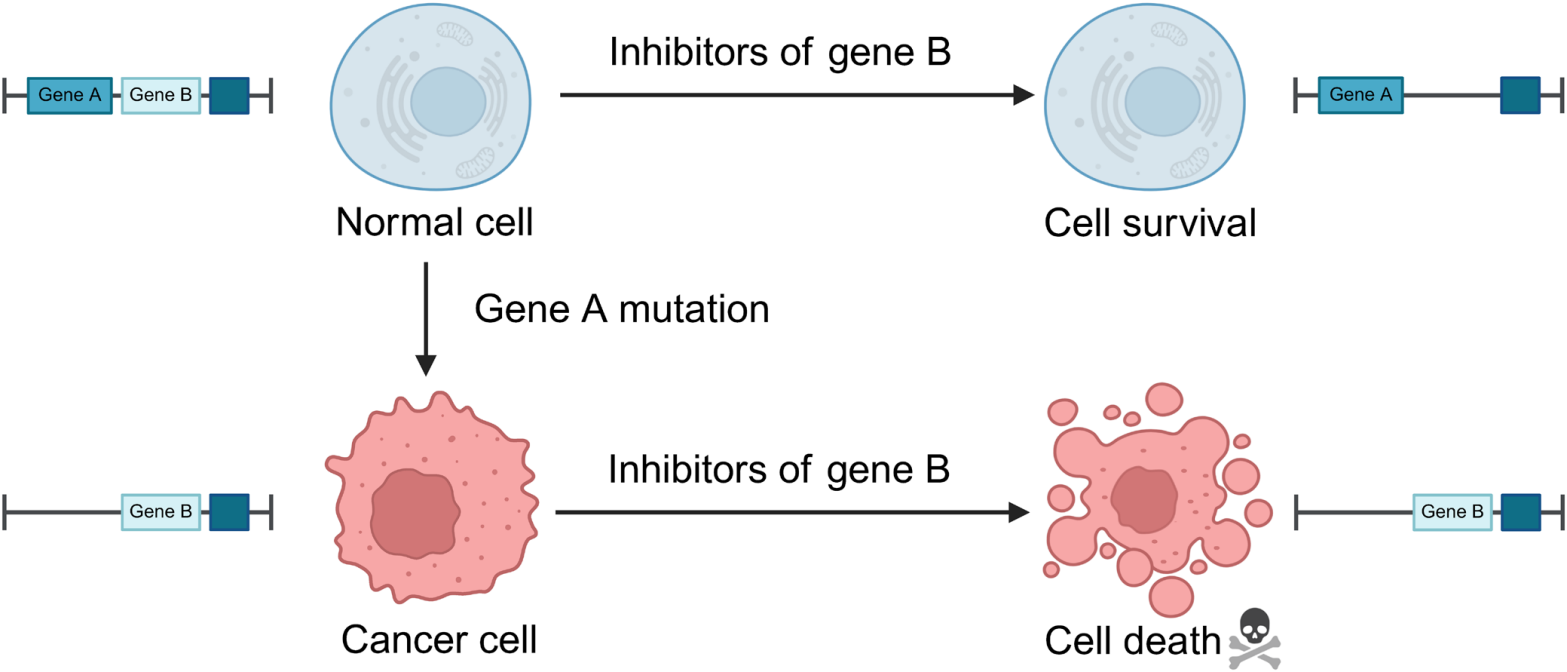
The principle of synthetic lethality. The defect, mutation or inhibition of either gene A or gene B alone does not compromise cell viability. However, pharmacologic inhibition of gene B in cells with a mutation in gene A induces simultaneous alterations in the proteins encoded by both genes. This process culminates in cell death via the mechanism of synthetic lethality. Created with BioRender.com.

In oncology research, the concept of synthetic lethality (SL) has demonstrated substantial potential for advancing cancer treatment [1]. First introduced in 1922 in a study on *Drosophila* hybridisation [2], synthetic lethality describes a unique biological phenomenon wherein simultaneous mutations or defects in a pair of genes result in cell death, while individual mutations in either gene have minimal effects on cell viability (Figure 1). The landmark study by Hartwell et al. marked the application of synthetic lethality in cancer treatment research [3]. This discovery not only illuminated the collaborative role of genes in controlling cellular phenotypes but also provided critical insights for optimising cancer drug discovery and development.

Targeted therapies, central to precision oncology, are designed to inhibit cancer driver genes based on the molecular characteristics of tumour cells [4, 5]. However, many potential cancer driver genes remain ‘undruggable’ [6], presenting a significant challenge. The concept of synthetic lethality offers a viable solution: genes that form a synthetic lethal relationship with these driver genes can serve as alternative targets, allowing for the precise targeting of tumour cells [7] and aligning with the goals of precision cancer therapy.

Over the past decades, synthetic lethality research has transitioned from foundational genetic studies to an interdisciplinary focal point, driven by technological advances in genomics, proteomics, and bioinformatics. In particular, the CRISPR–Cas9 system has enabled precise genome editing using single‐guide RNA (sgRNA), facilitating genome‐wide screening for synthetic lethal gene pairs [8]. These technological innovations have deepened our understanding of gene mutations and signalling networks in tumour cells [9], enabling the accurate identification and exploitation of synthetic lethal relationships and forming the basis for developing novel anticancer strategies.

Today, research on synthetic lethality has progressed from theoretical validation to the clinical testing of various synthetic lethal drugs. The first approved synthetic lethal drug, a PARP inhibitor targeting poly ADP‐ribose polymerase (PARP), has demonstrated efficacy against cancer cells with specific genetic mutations, such as BRCA1 or BRCA2 mutations [10]. As understanding of synthetic lethality theory expands, its application in clinical anticancer strategies continues to grow. This review aims to examine the application and evolution of synthetic lethality in tumour treatment, explore the discovery of novel targets, and investigate the integration of related drugs with existing cancer therapies. It highlights the clinical potential of synthetic lethality drugs, identifies emerging research trends, and underscores their anticipated role in providing more effective treatment options for cancer patients.

## 2 Synthetic Lethality

### 2.1 PARP

The BRCA1 and BRCA2 genes, located on chromosomes 17 and 13 respectively, are tumour suppressor genes involved in the homologous recombination repair (HRR) process during DNA damage repair. Mutations in these genes significantly increase the risk of breast and ovarian cancer. Statistics indicate that the risk of breast and ovarian cancer in BRCA1 mutation carriers is 57% and 40%, respectively, whereas the corresponding risks for BRCA2 mutation carriers are 49% and 18% [11]. Consequently, BRCA1/2 gene alterations are critical not only for monitoring breast and ovarian cancer risk but also for therapeutic interventions targeting these cancer types.

Poly (ADP‐ribose) polymerase (PARP) is a family of enzymes in eukaryotic cells that catalyse ADP‐ribosylation of themselves or target proteins [12]. The PARP family comprises 18 members, with PARP‐1 being the most abundant and responsible for over 90% of cellular ADP‐ribosylation activity [13]. PARP‐1, the most extensively studied member of this family, consists of three domains: the catalytic domain (CAT) at the C‐terminus, the automodification domain, and the N‐terminal DNA‐binding domain (DBD).

**FIGURE 2:**
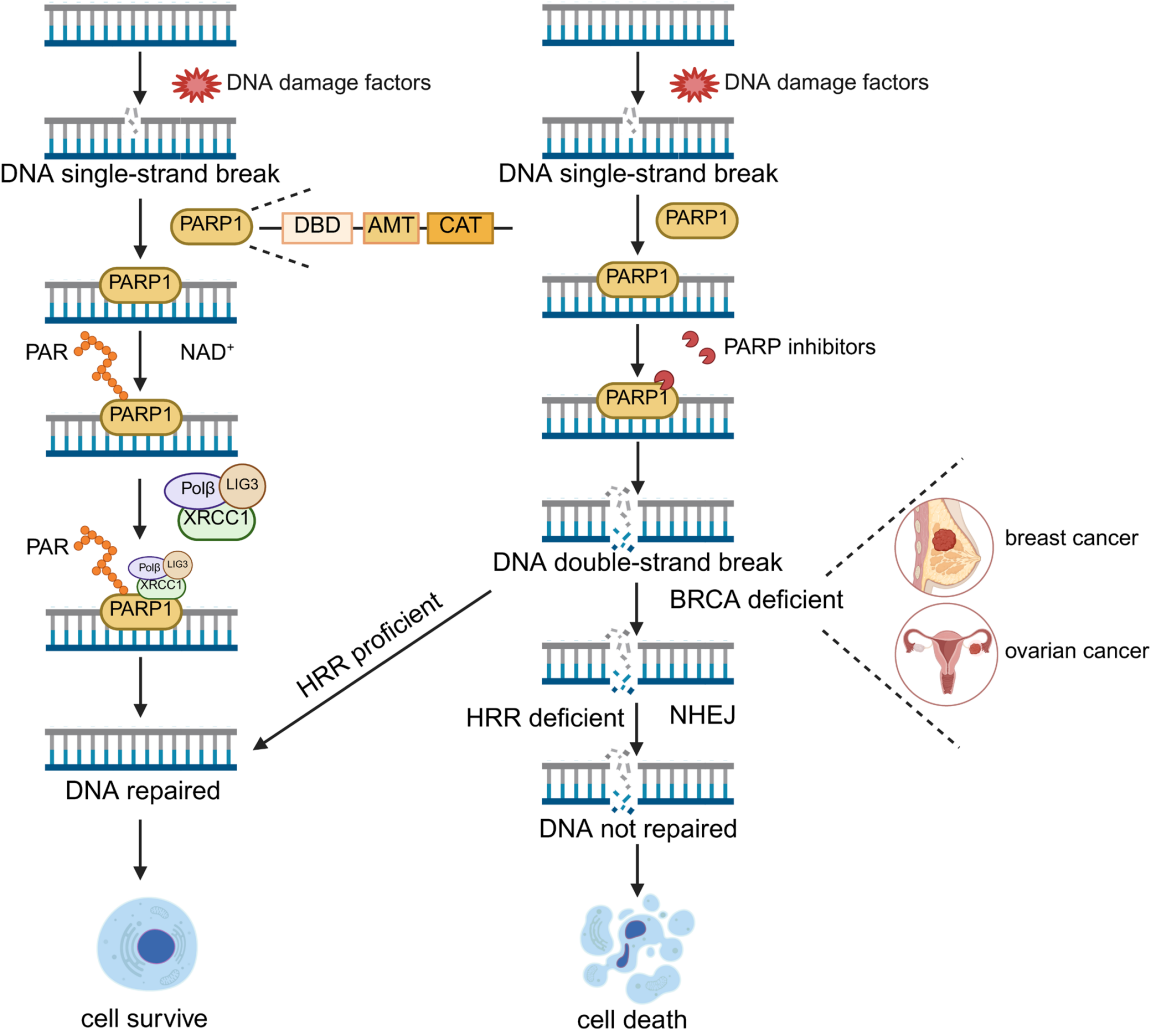
The synthetic lethal interaction between PARP and BRCA and the mechanisms of PARP inhibitor. Single‐strand breaks (SSBs) caused by various DNA‐damaging factors are typically repaired by PARP‐1. Upon binding to the damage site, PARP‐1 becomes activated. Once activated, PARP‐1 utilises NAD+ as a substrate to synthesise poly(ADP‐ribose) chains, resulting in structural modifications of nuclear protein receptors. This activity also facilitates the recruitment of downstream DNA repair proteins, such as x‐ray cross‐complementing group 1 (XRCC1). XRCC1, in turn, attracts DNA polymerase β (Polβ) and DNA ligase III (LIG3) to repair the DNA damage. The application of PARP inhibitors disrupts this repair pathway, leading to the accumulation of unrepaired SSBs, which can subsequently progress into double‐strand breaks (DSBs). In cells with a proficient homologous recombination repair (HRR) pathway, these DSBs can be effectively repaired. However, in BRCA‐deficient cells, the HRR pathway is compromised. Consequently, these cells rely on error‐prone non‐homologous end joining (NHEJ) for DNA repair, which ultimately results in genomic instability and cell death. Created with BioRender.com.

In 1980, it was observed that cellular levels of NAD+ decreased upon exposure to chemotherapeutic agents or ionising radiation, while PARP activity increased, underscoring its role in DNA damage repair [14]. PARP‐1 primarily participates in the base excision repair (BER) pathway, a component of the DNA damage response (DDR). It binds to single‐strand DNA breaks (SSBs) via its DNA‐binding domain, initiating activation. Upon activation, PARP‐1 utilises NAD+ as a substrate to synthesise poly (ADP‐ribose) chains while releasing nicotinamide. The negatively charged poly (ADP‐ribose) chains bind to nuclear protein receptors, altering their conformation and recruiting downstream DNA repair proteins such as XRCC1 (X‐ray cross‐complementing group 1) to the damage site [15], thereby facilitating DNA repair (Figure 2).

As PARP plays a critical role in DNA damage response (DDR), PARP inhibitors have been developed to induce tumour cell death by disrupting the DNA repair pathway. Early studies suggested the ‘DNA trapping’ mechanism to explain the action of PARP inhibitors. According to this mechanism, PARP inhibitors trap PARP at sites of DNA damage, inducing a conformational change that leads to irreversible DNA‐PARP binding. The persistence of the DNA‐PARP complex obstructs subsequent replication processes [15]. However, recent research has identified limitations in the DNA trapping mechanism and proposed an alternative explanation: PARP inhibitors exert their effects by inducing transcription–replication conflicts (TRC).

In eukaryotic cells, the TIMELESS‐TIPIN protein complex is a component of the fork protection complex (FPC) and is essential for DNA replication and repair, particularly in safeguarding the replication fork from transcriptional interference. Studies have shown that PARP1 interacts with TIMELESS, and together, they operate through the same molecular pathway to prevent TRC and mitigate DNA damage [16].

In 2005, researchers reported the synthetic lethal relationship between PARP inhibitors and BRCA1/2 mutations, offering a novel therapeutic approach for patients with BRCA1/2‐mutant tumours [17]. Under normal conditions, PARP inhibitors block the base excision repair (BER) pathway, leading to the accumulation of unrepaired single‐strand breaks (SSBs). These SSBs eventually result in double‐strand breaks (DSBs), which are typically repaired by the homologous recombination repair (HRR) pathway. However, tumour cells with BRCA1/2 mutations exhibit homologous recombination repair deficiency (HRD), relying instead on the error‐prone non‐homologous end joining (NHEJ) repair pathway. The resulting genetic instability causes cell cycle arrest and apoptosis, exemplifying the synthetic lethal effect between PARP inhibitors and BRCA1/2 mutations (Figure 2). Experimental evidence has further demonstrated that BRCA1/2‐mutant tumour cells are approximately 1000 times more sensitive to PARP inhibitors than wild‐type BRCA cells [17].

Since 2014, several PARP inhibitors—including olaparib, rucaparib, niraparib, talazoparib, fluzoparib, and pamiparib—have been approved for clinical use, achieving remarkable outcomes in the treatment of BRCA‐mutant tumours. Presently, the application of PARP inhibitors extends beyond the synthetic lethality associated with BRCA1/2 mutations to encompass a broader spectrum of HRD‐related gene mutations. This expansion suggests that the therapeutic scope of PARP inhibitors is broadening to include other tumour types sensitive to these inhibitors due to HRD [18].

Additionally, the aforementioned connection between PARP and TIMELESS‐TIPIN proteins has garnered significant research interest. Researchers are now investigating whether a synthetic lethal interaction can be established between the deletion of TIMELESS or TIPIN and HRD. It has been observed that co‐deletion of BRCA2 with TIMELESS or TIPIN induces genetic instability in HeLa cells and ultimately leads to cell death. This finding highlights the potential of TIMELESS and TIPIN as novel targets for synthetic lethality [16].

### 2.2 ATR

The transduction of DNA damage signals, akin to other cellular signalling cascades, is mediated by protein phosphorylation, involving members of the phosphoinositide 3‐kinase‐related kinase (PIKK) family. These include DNA‐dependent protein kinase catalytic subunits (DNA‐PKcs), ataxia telangiectasia mutated (ATM), and ataxia telangiectasia and Rad3‐related protein (ATR) [19]. ATM and DNA‐PKcs primarily respond to DNA double‐strand breaks (DSBs), whereas ATR is implicated in the response to genotoxic stress.

ATR consists of 2644 amino acids, with a kinase domain at the C‐terminus that phosphorylates downstream target proteins and an ATR‐interacting protein (ATRIP)‐binding domain at the N‐terminus, which activates ATR. When cellular DNA experiences damage or replication stress, ATR binds to ATRIP and is recruited to single‐stranded DNA (ssDNA) coated with replication protein A (RPA). Topoisomerase‐binding protein 1 (TopBP1) and Ewing sarcoma‐associated tumour antigen (ETAA1) assist in ATR activation [20]. TopBP1 interacts with the RAD9‐RAD1‐HUS1 (9‐1‐1) complex, which, with the aid of the RAD17‐RFC complex, localises to DNA damage sites. ETAA1, in contrast, directly binds RPA‐coated ssDNA to activate ATR. ATR undergoes autophosphorylation at T1989, activating cell cycle checkpoint kinase 1 (Chk1), which promotes the degradation of cell division cycle 25 (CDC25). As CDC25 is a phosphatase that removes inhibitory modifications from cyclin‐dependent kinases (CDKs), ATR‐mediated CDC25 degradation inhibits CDK activity, leading to cell cycle arrest and allowing time for DNA repair before mitosis [21].

Moreover, during replication stress, the helicase often continues to operate while DNA polymerase ceases functioning, leading to the formation of stalled replication forks. At this DNA damage site, if the helicase SMARCAL1 is aberrantly activated, it can produce nucleic acid substrates susceptible to cleavage by SLX4 proteins, potentially resulting in extensive accumulation of DSBs and ssDNA templates.

**FIGURE 3:**
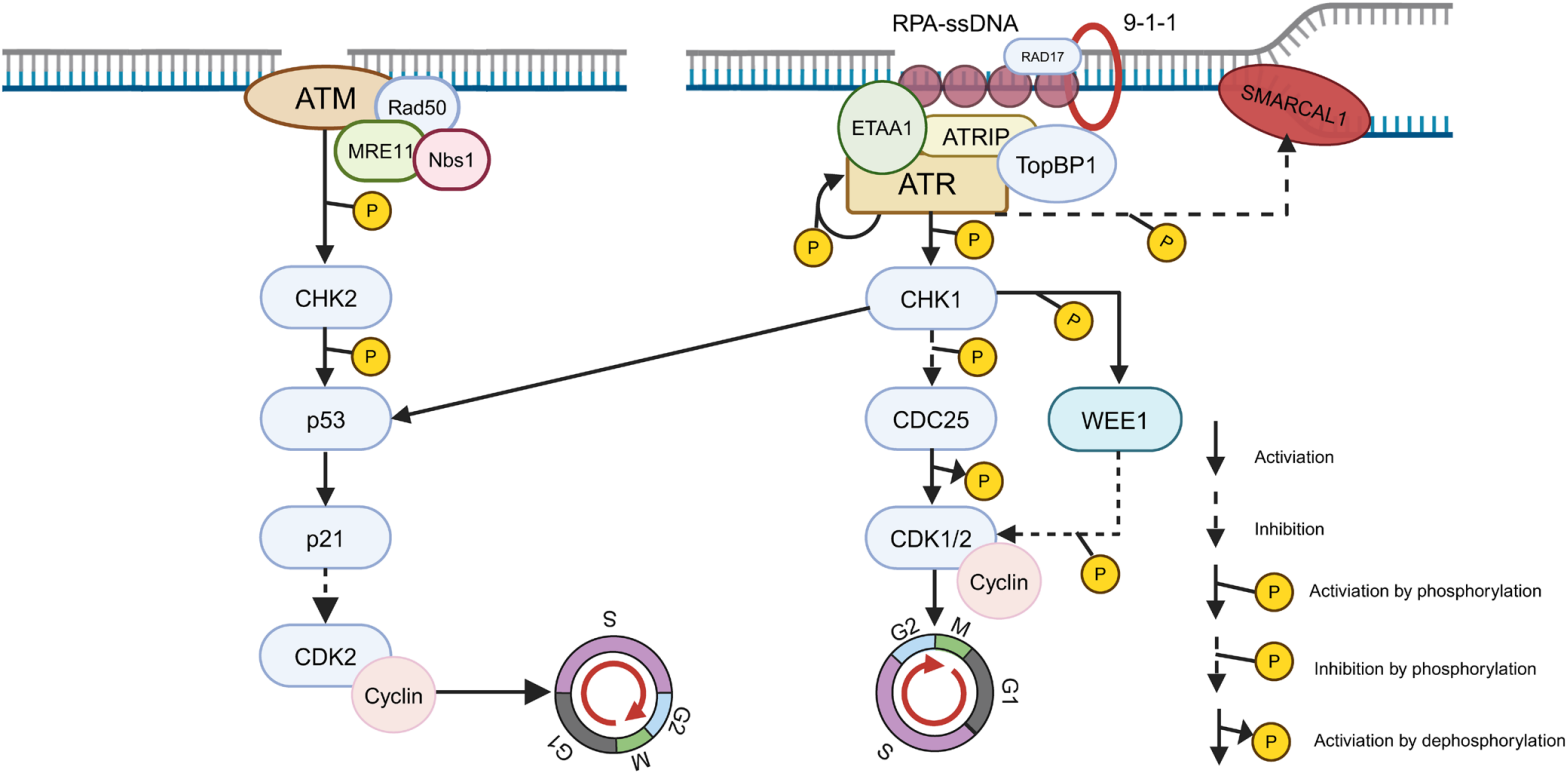
The mechanisms of ATM, ATR and WEE1 in cell cycle arrest. ATM is recruited and activated by the MRE11‐RAD50‐NBS1 complex (MRN complex) on double‐strand breaks (DSBs). ATM signalling subsequently triggers the activation of the CHK2 kinase, which phosphorylates and stabilises the tumour suppressor p53. As a transcription factor, p53 binds to the promoter region of the p21 gene, thereby activating p21 transcription. This leads to the inhibition of CDK2 activity and arrests the cell cycle at the G1/S checkpoint. ATR, in conjunction with ATRIP, binds to single‐stranded DNA (ssDNA) wrapped with RPA, TopBP1, and ETAA1 to facilitate ATR's activation. ATR then phosphorylates itself and CHK1, initiating a signalling cascade. Concurrently, ATR inhibits SMARCAL1 to halt the progression of the replication fork. The activation of CHK1 subsequently promotes the degradation of CDC25, which dephosphorylates and activates CDK1/2. CHK1 also activates WEE1, which further inhibits the activity of CDK1/2. These reactions collectively arrest the cell cycle at the G2/M checkpoint by inhibiting CDK, allowing time for DNA repair. Notably, in addition to CDC25 and WEE1, CHK1 can also activate p53, serving as a crossover point between ATM and ATR signalling pathways. Created with BioRender.com.

To preserve genomic stability, ATR inhibits this process via dual mechanisms: firstly, it directly phosphorylates SMARCAL1 to suppress its helicase activity and avert the production of aberrant structures [22] (Figure 3); secondly, it phosphorylates Rad18, disrupting its interaction with PCNA and thereby inhibiting PCNA monoubiquitination. This signalling cascade is the critical pathway for recruiting SLX4 to stalled replication forks [23]. This regulation maintains SLX4 in a ‘controlled state’ during replication fork stabilisation, thereby preventing premature remodelling or abnormal processing of replication forks. However, the DNA damage repair function of SLX4 is activated when encountering sustained replication stress or the occurrence of interstrand crosslinks (ICLs). Acting as a molecular scaffold, SLX4 recruits structure‐selective endonucleases (SSEs), including SLX1, MUS81‐EME1, and XPF‐ERCC1, to precisely cleave barrier structures such as Holliday junctions [24]. Especially when replication forks are impeded by tight DNA‐protein complexes, SLX4‐XPF‐mediated targeted cleavage generates ssDNA, which in turn recruits ATR to amplify repair signals and maintain genomic stability [25]. In conclusion, SLX4 and ATR play a balanced and coordinated role in ensuring the stability and integrity of the genome.

Tumour cells, often defective in DNA repair pathways, are more reliant on functional DNA repair mechanisms than normal cells. Additionally, due to their elevated replication stress, tumour cells exhibit enhanced dependence on the ATR signalling pathway. Experimental evidence has shown that ATR expression and activation are closely correlated with tumour stage, mitotic index, vascular invasion, and patient prognosis [26, 27]. Consequently, ATR has emerged as a promising target in cancer therapy, with researchers investigating its synthetic lethal potential in a manner analogous to the relationship between PARP inhibitors and HRD.

The tumour suppressor protein p53 is stabilised via ATM‐mediated phosphorylation during DDR. ATM activation promotes p53's transcriptional activity, leading to the upregulation of genes such as p21, which induces G1 phase cell cycle arrest. Solid tumour cells with ATM loss or p53 mutations exhibit heightened sensitivity to ATR inhibitors, demonstrating a synthetic lethal effect between ATR inhibitors and defects in the ATM‐p53 pathway [28, 29].

To date, numerous ATR inhibitors, including VX‐970, AZD6738, BAY1895344, ATR0380, and RP‐3500, have entered clinical trials and shown substantial promise in phase I and phase II studies (Table 1).

**TABLE 1:** Clinical trials of ATR inhibitors for cancer treatment.

Drug	Health conditions	NCT number	Phases	Status
Berzosertib (VX‐970)	Small cell lung cancer, extra‐pulmonary small cell neuroendocrine cancer	NCT04826341	I/II	Recruiting
	Small cell cancer	NCT04802174	I/II	Recruiting
	Leiomyosarcoma	NCT04807816	II	Recruiting
	Small cell lung cancer	NCT04768296	II	Completed
	Lung non‐small cell squamous carcinoma	NCT04216316	I/II	Active
	Solid tumours	NCT04266912	I/II	Active
	Breast cancer	NCT04052555	I	Active
	Small cell cancer	NCT03896503	II	Active
	Solid tumours	NCT03718091	II	Completed
	Gastric cancer	NCT03641313	II	Active
	Prostate cancer	NCT03517969	II	Active
	Solid tumours	NCT02723864	I	Active
	Ovarian cancer, peritoneal cancer or fallopian tube cancer	NCT02595892	II	Active
	Head and neck cancer	NCT02567422	I	Active
Ceralasertib (AZD6738)	Non‐small lung cancer	NCT05941897	II	Active
	Breast cancer	NCT05582538	II	Recruiting
	Solid tumours	NCT05514132	I	Active
	Chronic lymphocytic leukemia	NCT05404282	I	Active
	Solid tumours	NCT04704661	I	Recruiting
	Small cell lung cancer	NCT04699838	II	Recruiting
	Osteosarcoma	NCT04417062	II	Recruiting
	Breast cancer	NCT04090567	II	Recruiting
	Gynaecological cancer	NCT04065269	II	Recruiting
	Solid tumours	NCT03682289	II	Recruiting
	Solid tumours	NCT02223923	I	Active
Tuvusertib (M1774)	Epithelial ovarian cancer	NCT06433219	II	Recruiting
	Lung non‐small cell squamous carcinoma	NCT05882734	I/II	Active
	Prostate cancer	NCT05828082	II	Recruiting
	Colorectal cancer	NCT05691491	I/II	Recruiting
	Solid tumours	NCT05687136	I	Recruiting
Elimusertib (BAY1895344)	Solid tumours	NCT05071209	I/II	Active
	Pancreatic cancer, ovarian cancer	NCT04616534	I	Active
	Head and neck cancer	NCT04576091	I	Active
	Colorectal cancer, gastric cancer	NCT04535401	I	Active
	Small cell lung cancer, neuroendocrine cancer, pancreatic cancer	NCT04514497	I	Active
	Solid tumours	NCT04491942	I	Active
	Ovarian cancer	NCT04267939	I	Terminated
	Solid tumours	NCT04095273	I	Completed
ART0380	Solid tumours	NCT05798611	II	Recruiting
	Solid tumours	NCT04657068	I/II	Recruiting
Camonsertib (RP‐3500)	Solid tumours	NCT04972110	I/II	Active
	Solid tumours	NCT04497116	I/II	Recruiting

*Note:* These clinical trial data are from ClinicalTrials.gov (June 2025).

### 2.3 WEE1

WEE1 kinase, a member of the WEE family, is a serine/threonine protein kinase that plays a critical role in the regulation of the cell cycle, particularly during DNA replication and repair. It is highly conserved across eukaryotic organisms. WEE1 is active during the S and G2 phases of the cell cycle, especially at the crucial G2/M checkpoint, where it ensures the completion of DNA replication and the repair of DNA damage to maintain genomic stability before mitotic entry. When DNA damage occurs, ATR activates WEE1 kinase via the downstream phosphorylation of cell cycle checkpoint kinase 1 (Chk1), which, in turn, phosphorylates WEE1 [30].

Activated WEE1 phosphorylates the Tyr15 site of CDK1 in the CDK1/cyclin B complex, inhibiting CDK1 activity and arresting the cell cycle at the G2 phase. This arrest provides the cell with sufficient time for DNA repair (Figure 3). Additionally, WEE1 has been shown to influence histone synthesis. During the S phase, WEE1 binds to and phosphorylates histone H2B, thereby regulating histone synthesis to maintain an appropriate ratio between newly synthesised DNA and histones [31]. This precise balance is essential for preserving chromosomal stability. Consequently, WEE1 is recognised as a key regulator of genomic integrity.

Under normal circumstances, DNA damage activates two key cell cycle checkpoints—G1/S and G2/M—to ensure accurate progression into mitosis. However, tumour cells frequently exhibit defects in the G1/S checkpoint [32], such as mutations in the p53 gene, rendering them heavily reliant on the G2/M checkpoint for DNA repair. Inhibition of WEE1 under these conditions induces synthetic lethality, as tumour cells lose the ability to repair DNA damage, prematurely enter mitosis, and ultimately undergo cell death. Studies have demonstrated that the application of WEE1 inhibitors specifically induces death in p53‐deficient tumour cells [33, 34].

Moreover, WEE1 expression is elevated in various malignancies, including glioblastoma [35], malignant melanoma [36], pancreatic cancer [37], and triple‐negative breast cancer [38], with its expression level closely correlated with patient prognosis. Consequently, investigating the mechanisms of WEE1 in these tumours and developing WEE1 inhibitors have become pivotal focuses of cancer therapy. Several WEE1 inhibitors are currently under development, including adavosertib, azenosertib, and debio 0123. Among these, adavosertib was the first WEE1 inhibitor to enter clinical trials [39], while azenosertib, a more selective WEE1 inhibitor, represents a cutting‐edge development in WEE1‐targeted therapies (Table 2).

**TABLE 2:** Clinical trials of WEE1 inhibitors for cancer treatment.

Drug	Health conditions	NCT number	Phases	Status
Adavosertib (AZD1775)	Uterine serous cancer	NCT04590248	II	Completed
	Solid tumours	NCT04462952	I	Completed
	Oesophageal and gastroesophageal junction cancer	NCT04460937	I	Active
	Solid tumours	NCT04197713	I	Active
	Ovarian cancer, peritoneal cancer or fallopian tube cancer	NCT03579316	II	Active
	Cervical, upper vaginal and uterine cancers	NCT03345784	I	Active
	Solid tumours	NCT03284385	I	Active
	Ovarian cancer, peritoneal cancer or fallopian tube cancer	NCT02659241	II	Active
Azenosertib (ZN‐c3)	Gastric cancer	NCT06364410	I	Recruiting
	Pancreatic cancer	NCT04005690	I	Recruiting
Debio 0123	Small cell lung cancer	NCT05815160	I	Recruiting
	Solid tumours	NCT05109975	I	Recruiting

*Note:* These clinical trial data are from ClinicalTrials.gov (June 2025).

### 2.4 WRN

The DNA mismatch repair (MMR) mechanism is a highly conserved pathway in prokaryotic and eukaryotic organisms that plays a vital role in maintaining genome integrity. By recognising and correcting base mismatches introduced by DNA polymerase errors or environmental factors during replication, MMR ensures accurate genetic transmission [40]. Key proteins involved in this process include MSH2, MSH3, MSH6, MLH1, and PMS2, which form heterodimers such as MutS and MutL, maintaining the proper function of the MMR mechanism [41].

Short tandem repeats, known as ‘microsatellites’, are genomic regions composed of 1–6 nucleotide repeats that occupy 1%–3% of the human genome [42]. During replication, DNA polymerase frequently slips in these regions, leading to mismatches that are heavily reliant on the MMR system for repair [43]. In the absence of a functional MMR system, these errors result in insertion or deletion mutations in microsatellite sequences, producing a phenomenon known as microsatellite instability (MSI). MSI is observed in approximately 5%–15% of patients with metastatic colorectal cancer [44]. MMR deficiency and high MSI (MSI‐H) status have become integral components of colorectal cancer diagnosis [45].

Werner syndrome helicase (WRN) belongs to the RecQ family of helicases and is unique within the family due to its dual helicase and 3′–5′ exonuclease activities. WRN comprises four structural domains: the N‐terminal exonuclease domain, the ATPase domain, the RecQ C‐terminal (RQC) domain that binds DNA, and the helicase‐ribonuclease D C‐terminal (HRDC) domain. This dual enzymatic capability enables WRN to process various DNA structures, including double‐stranded DNA, D‐loops, G‐quadruplexes, and cruciform structures [46, 47].

**FIGURE 4:**
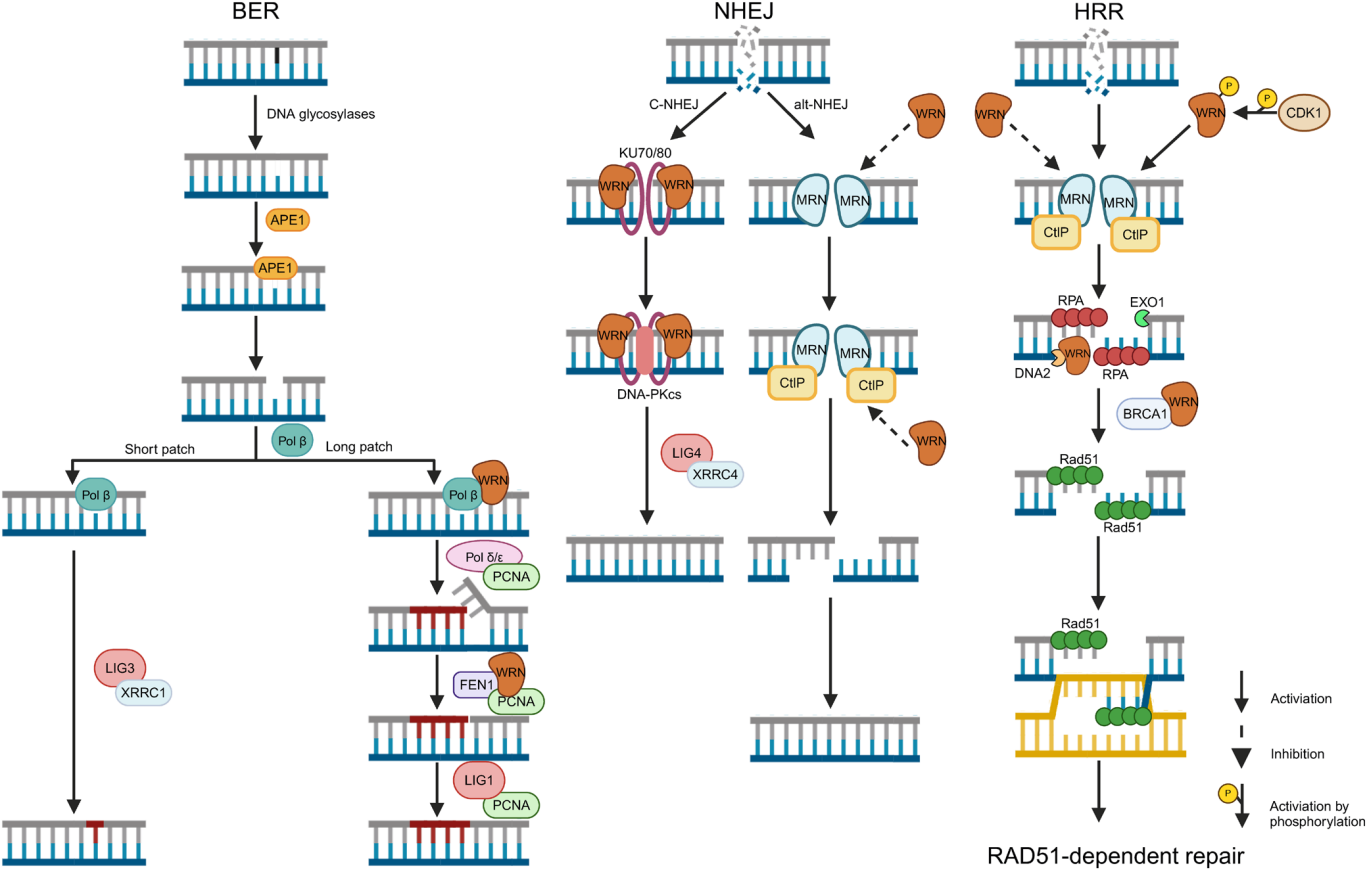
The role of WRN in DNA damage repair. WRN is involved in multiple biological activities during genome maintenance. In the BER pathway, WRN mainly participates in the long‐patch BER pathway, where it interacts with DNA polymerase β (Polβ), flap endonuclease 1 (FEN1) and proliferating cell nuclear antigen (PCNA). When confronted with double‐strand breaks (DSBs), WRN makes a decision among HRR, c‐NHEJ, and alt‐NHEJ. Among these pathways, WRN prefers c‐NHEJ. WRN inhibits the MRE11‐RAD50‐NBS1 complex (MRN complex) and the C‐terminal binding protein interacting protein (CtIP), both of which are integral to alt‐NHEJ and HRR processes. CDK1 can phosphorylate WRN to reverse its inhibitory effect on MRN and CtIP. Conversely, in c‐NHEJ, WRN not only physically interacts with the Ku70/80 complex but is also activated by this complex. WRN's exonuclease activity is critical for DSB repair because it can process DNA ends to generate substrates suitable for the XRCC4‐DNA ligase IV complex. Furthermore, in the HRR pathway, WRN interacts with DNA2 and BRCA1 to process and resect DNA ends. Created with BioRender.com.

In double‐strand break (DSB) repair, WRN acts as a molecular switch, modulating the choice between homologous recombination (HR) and non‐homologous end joining (NHEJ) through distinct phosphorylation states in conjunction with the RAD51 protein [48]. During NHEJ, WRN preferentially promotes classical NHEJ (c‐NHEJ) while inhibiting the alternative NHEJ (alt‐NHEJ) pathway. WRN also suppresses the activity of the MRE11‐RAD50‐NBS1 (MRN) complex and C‐terminal binding protein interacting protein (CtIP), factors involved in DNA end resection during HR, to prevent excessive DNA degradation and preserve genome stability [49]. Additionally, WRN interacts synergistically with key proteins in the base excision repair (BER) pathway [50] (Figure 4). Thus, WRN is implicated in various processes essential for genome maintenance, including DNA damage repair, telomere stabilisation, and autophagy [51].

In recent years, several studies [52, 53, 54, 55] have demonstrated that tumour cells deficient in mismatch repair (MMR), particularly those exhibiting microsatellite instability (MSI), undergo cell death upon knockdown of the WRN gene. Conversely, WRN is not essential for the survival of microsatellite‐stable (MSS) cell lines [53]. Notably, the helicase domain of WRN, rather than the exonuclease domain, is critical for the survival of MSI cells. Persistent MSI results in the amplification of TA nucleotide repeat sequences and the formation of cruciform DNA structures, which lead to replication arrest. WRN, recruited and activated by ATR kinase, unwinds these cruciform structures [56], thereby preventing double‐strand breaks (DSBs). In the absence of WRN, unresolved replication fork arrest caused by these structures persists until mitosis, where intermediates are cleaved by MUS81 nuclease and SLX4 protein, resulting in DSBs that ultimately cause cell death [57].

The dependency of MSI cells on WRN highlights WRN as a novel target for synthetic lethality. Differences exist in the synthetic lethal interactions between WRN and MSI and those between PARP and BRCA. The sensitivity of BRCA‐mutant tumour cells to PARP inhibitors is associated with sustained homologous recombination (HR) deficiencies, and resistance to PARP inhibitors arises through HR restoration. However, WRN dependence is specific to MSI rather than multiple MMR mutations, and the relationship between TA‐amplified regions in MSI and MMR mutations remains unclear. It is hypothesised that the cellular dependency on WRN originates from MSI rather than directly from MMR mutations. Furthermore, while functional restoration of the MMR system partially mitigates the lethal effects of WRN loss in MSI cells, this reversal is not complete.

The most rapidly advancing WRN inhibitors include HRO761, a non‐covalent inhibitor, and VVD‐133214, a covalent inhibitor that selectively binds to the C727 site on WRN [58, 59].

## 3 Combined Strategies of Synthetic Lethality and Chemotherapy

Therapeutic targets identified based on the principle of synthetic lethality and the corresponding novel drugs have emerged as innovative strategies for targeted therapies and for enhancing the sensitivity of tumours to chemotherapeutic agents, marking a significant advancement in the era of precision medicine. Commonly used chemotherapeutic agents can be broadly categorised as follows: Alkylating agents induce DNA cross‐linking, disrupting transcription and replication [60]. Antimetabolites inhibit the synthesis of folic acid, purines, and pyrimidines, essential for DNA synthesis [61]. Topoisomerase inhibitors block enzymes that relieve DNA supercoiling during replication and transcription [62]. Other categories include platinum‐based compounds, antitumour antibiotics, phytochemicals, hormones, and others. The combination of synthetic lethality‐based drugs with the aforementioned chemotherapeutic agents can enhance the cytotoxic effect on tumour cells while minimising damage to normal cells, which are increasingly being applied in clinical practice.

PARP inhibitors, as the first approved synthetic lethal drugs for clinical use, have been extensively studied in combination with conventional chemotherapeutics. In ovarian cancer treatment, results from the SOLO1 [63, 64] and PRIMA [65] clinical trials demonstrated the efficacy of combining PARP inhibitors with platinum‐based chemotherapies. The SOLO1 trial evaluated the use of the PARP inhibitor olaparib as maintenance therapy for newly diagnosed, BRCA‐mutated advanced ovarian cancer (AOC). The 5‐year follow‐up [63] revealed a median progression‐free survival (PFS) of 56.0 months in the olaparib group versus 13.8 months in the placebo group. Kaplan–Meier analysis showed a 5‐year progression‐free rate of 48.3% in the olaparib group, significantly higher than the 20.5% in the placebo group. With up to 7 years of follow‐up [64], the overall survival (OS) hazard ratio was 0.55, although OS data were not yet mature. According to Kaplan–Meier estimates, 67.0% of patients in the olaparib group survived at 7 years, compared with 46.5% in the placebo group, suggesting that olaparib significantly reduced the risk of death.

PRIMA trial focused on evaluating the efficacy of niraparib in different genetic backgrounds and included homologous recombination repair (HRR) status as the classification standard. The latest results [65] showed that the PFS hazard ratios of the niraparib group in the overall cohort, homologous recombination deficiency (HRD) and homologous recombination proficient (HRP) subgroups were 0.66, 0.51, and 0.67, respectively, indicating that niraparib can significantly prolong PFS of patients, but in terms of OS, the hazard ratios of each population were close to 1, which did not reach statistical significance.

Moreover, the PAOLA1 [66] trial investigated whether combining olaparib with the antiangiogenic agent bevacizumab could further improve PFS and OS in AOC. The results indicated that the median OS was significantly extended with the olaparib‐bevacizumab combination in patients with HRD‐positive and BRCA‐mutant tumours, reaching 75.2 months versus 57.3 months and 75.2 months versus 66.9 months, respectively. However, this combination did not yield a significant OS benefit in the overall cohort of AOC patients or in those with HRD‐positive but BRCA wild‐type tumours. The 5‐year PFS data further elucidated the combination's efficacy: in the overall AOC population, the median PFS was 22.9 months for the olaparib‐bevacizumab group compared to 16.6 months for the bevacizumab‐alone group. In HRD‐positive patients, the median PFS was 46.8 months versus 17.6 months; in BRCA‐mutant patients, it was 60.7 months versus 21.7 months; and in HRD‐positive but BRCA wild‐type patients, it was 30.0 months versus 16.6 months. These findings underscore the synergistic advantages of the olaparib‐bevacizumab combination in treating AOC, solidifying PARP inhibitors' role as a first‐line maintenance therapy for newly diagnosed patients with this disease.

In breast cancer, the BROCADE3 Phase III trial [67] evaluated the efficacy of veliparib combined with carboplatin and paclitaxel in HER2‐negative, BRCA‐mutated breast cancer. The median PFS (mPFS) was 14.5 months in the combination group, compared to 12.6 months in the group treated with carboplatin and paclitaxel alone. Regarding adverse events, the BROCADE3 Phase III trial [67] demonstrated that there were no significant differences in the incidence of the most common grade ≥ 3 adverse reactions (AEs), namely neutropenia and anaemia, between the veliparib monotherapy group and the combination therapy group when compared with the placebo group. Specifically, the incidence rates of neutropenia and anaemia were 3.7% in the veliparib group, whereas they were 5.2% and 1.7%, respectively, in the placebo group. However, during the veliparib combination treatment phase, the incidence of these AEs was significantly higher: 81.0% of patients in the veliparib group experienced neutropenia and 42% experienced anaemia, compared with 83.6% and 39.8%, respectively, in the placebo group. Additionally, three grade ≤ 2 seizure events were observed in the veliparib monotherapy group, while no such events occurred in the placebo group. Overall, veliparib not only enhanced the efficacy of traditional chemotherapy drugs and significantly improved progression‐free survival, but also did not significantly increase the risk of serious adverse events.

A Phase I clinical trial [68] investigated veliparib in combination with cyclophosphamide in advanced solid tumours, as well as its potential with cyclophosphamide and doxorubicin in breast cancer treatment. Additional studies showed that veliparib potentiated the cytotoxic effects of 5‐fluorouracil (5‐FU) on colorectal cancer stem cells by inhibiting the MMR pathway [69]. These findings underscore the potential of PARP inhibitors to enhance the efficacy of DNA‐damaging agents in combination therapies.

Several ATR inhibitors, including VX‐970 (M6620) and ceralasertib (AZD6738), are under active clinical investigation. Early trials frequently combine ATR inhibitors with chemotherapeutics that induce replication stress. ATR inhibitors have been shown to enhance cisplatin sensitivity and reduce resistance in tumour cells [70, 71]. Furthermore, combining AZD6738 with the antimetabolite gemcitabine significantly induced regression of pancreatic ductal adenocarcinoma [72]. Similarly, the combination of topotecan and ATR inhibitors has shown promise in treating small‐cell lung cancer [73]. WEE1 inhibitors also enhance the cytotoxic effects of DNA‐damaging agents. The WEE1 inhibitor MK‐1775 has demonstrated greater efficacy in p53‐deficient tumour cells when combined with agents such as gemcitabine, carboplatin, cisplatin or 5‐fluorouracil, compared to its use as a monotherapy [74, 75].

Despite the notable success of numerous clinical trials, the application of synthetic lethal drugs in combination with conventional chemotherapeutic agents presents significant challenges. For instance, the use of PARP inhibitors in conjunction with certain chemotherapeutic agents has been associated with overlapping toxicities, particularly myelosuppression, which may restrict the feasibility of these combinations [76]. Consequently, research efforts have increasingly focused on identifying alternative strategies to reduce toxicity while maintaining therapeutic efficacy.

## 4 The Combined Strategy of Synthetic Lethal Drugs and Radiotherapy

Radiotherapy (RT), one of the core modalities of tumour treatment, is administered to approximately 50% of cancer patients during their course of treatment and accounts for 4% of curative treatment options [77]. The antitumour effect of RT relies on inducing varying degrees of DNA damage through ionising radiation. However, the DNA damage response (DDR) in tumour cells rapidly detects and repairs these lesions [78]. Therefore, the efficacy of RT is contingent on ensuring that the degree of ionising radiation‐induced damage surpasses the cell's intrinsic repair capacity [79]. Blocking the DNA damage repair pathways is thus emerging as a key strategy for identifying radiosensitizers, with inhibitory drugs targeting DDR molecules playing a critical role in enhancing the efficacy and sensitivity of tumour cells to RT [80, 81].

PARP inhibitors, as specific inhibitors of the base excision repair pathway in the DDR, have demonstrated significant efficacy in combination with RT. This has been validated through extensive preclinical and clinical studies (Table 3), with their radiosensitising effect observed beyond BRCA mutations or homologous recombination deficiencies (HRD). In an in vitro study on cervical cancer [80], the combination of RT, cisplatin, and PARP inhibitors significantly outperformed monotherapy in suppressing tumour cell growth. Furthermore, in radioresistant cholangiocarcinoma [82], the combination of the PARP inhibitor olaparib with RT markedly reduced tumour cell proliferation, confirming the radiosensitising potential of olaparib.

**TABLE 3:** Clinical trials of PARP inhibitors combined with radiotherapy.

Drug	Health conditions	NCT number	Phases	Status
Niraparib	Ovarian cancer	NCT05990192	II	Recruiting
	Head and neck squamous cell carcinoma	NCT05784012	I/II	Recruiting
	Rectal cancer	NCT04926324	I/II	Suspended
	Breast cancer	NCT04837209	II	Recruiting
	Prostate cancer	NCT04037254	II	Active
Olaparib	Breast cancer	NCT04683679	II	Recruiting
	Breast cancer	NCT03598257	I	Active
	High grade gliomas	NCT03212742	I/II	Active
	Breast cancer	NCT03109080	I	Completed
	Soft‐tissue sarcoma	NCT02787642	I	Completed
Rucaparib	Breast cancer	NCT03542175	I	Active

*Note:* These clinical trial data are from ClinicalTrials.gov (June 2025).

Although the exact mechanisms underlying the radiosensitising effects of PARP inhibitors remain incompletely understood, several key factors have been identified. First, PARP inhibitors influence cell cycle redistribution, an important determinant of tumour cell resistance to RT [83] and a primary mechanism for their radiosensitising activity. Cellular sensitivity to RT varies across the cell cycle, with the G2/M phase being the most sensitive, followed by the G1 phase, and the S phase being the least sensitive. Olaparib has been shown to increase the proportion of cells in the G2/M phase, thereby enhancing tumour cell sensitivity to RT. Secondly, PARP inhibitors increase the incidence of DNA double‐strand breaks in tumour cells, leading to enhanced cell death [82].

The radiosensitivity of tumour cells is closely related to the oxygenation status of the tumour microenvironment. Radiotherapy uses ionising radiation to generate reactive oxygen species (ROS), which inflict damage on DNA, proteins, and other biomolecules, ultimately leading to cell damage and necrosis. However, the rapid proliferation characteristic of solid tumours frequently induces a hypoxic microenvironment. This hypoxia is a pivotal factor that curtails ROS production and consequently results in radioresistance [84]. PARP inhibitors influence tumour growth, metastasis, drug resistance, and overall treatment response by enhancing oxygenation within the tumour microenvironment. For instance, PARP inhibitors olaparib share structural similarities with nicotinamide but exhibit superior vasodilatory activity [85].

Under hypoxic conditions, the upregulation of hypoxia‐inducible factor 1 alpha (HIF‐1α) and vascular endothelial growth factor (VEGF) in cells drives the formation of tumour neovascularisation. These newly formed vessels, however, are often structurally abnormal and functionally defective, leading to poor oxygen transport efficiency [86]. Moreover, tumour tissues in hypoxic environments may develop vascular mimicry (VM), which refers to the formation of vessel‐like structures that can transport blood cells and immune cells but lack endothelial cells. The existence of VM is often positively correlated with the invasiveness of tumour cells [87].

**FIGURE 5:**
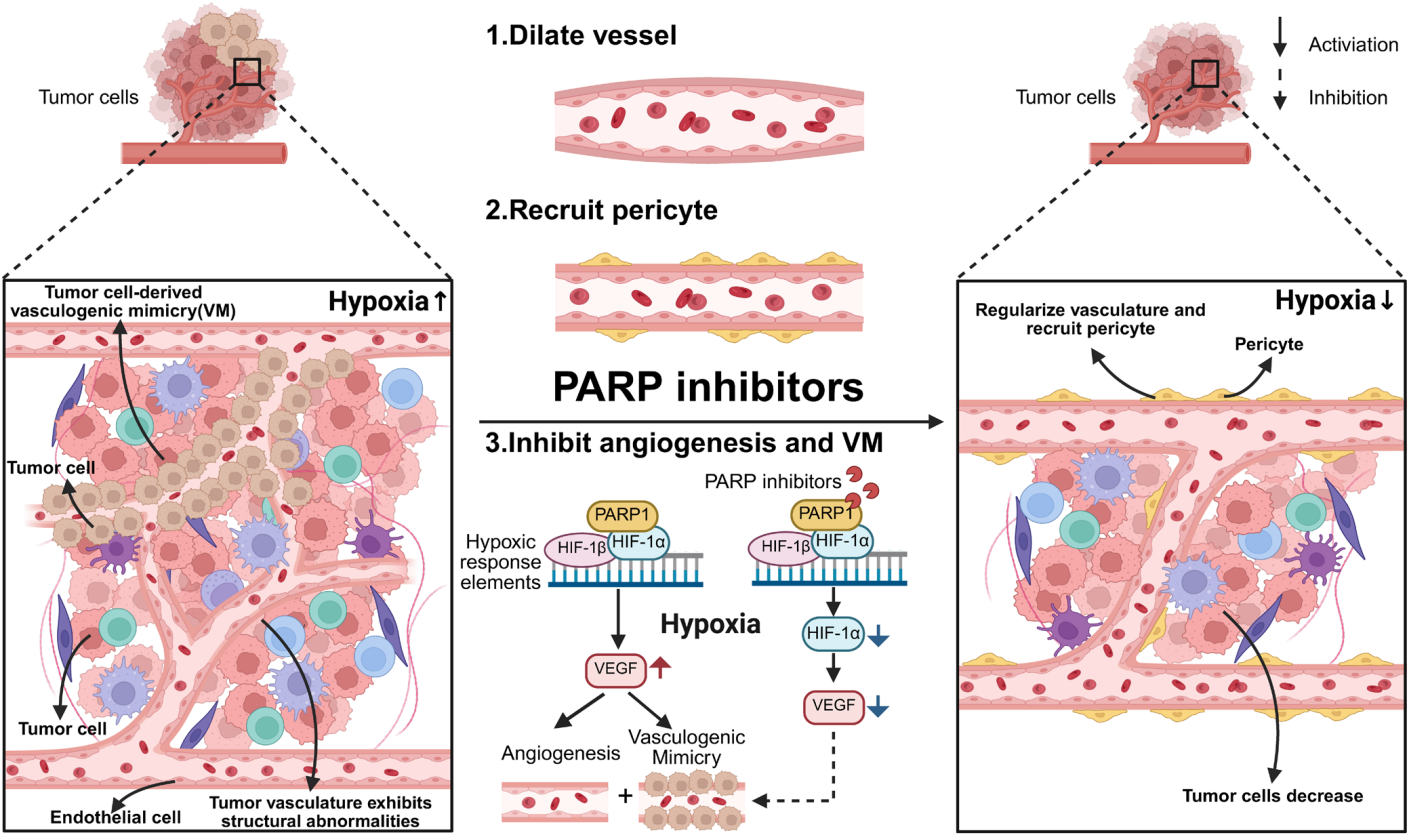
The role of PARP inhibitors in improving oxygenation in tumour microenvironment. Rapid tumour cell proliferation induces hypoxia within the tumour microenvironment (TME), thereby activating HIF and VEGF expression. This activation promotes both tumour angiogenesis and vascular mimicry (VM), a process where tumour cells form vessel‐like structures without endothelial cells to transport blood. However, these vessels are structurally abnormal, characterised by endothelial cell cracks and low oxygen transport efficiency. PARP inhibitors can increase oxygen levels of TME through multiple mechanisms. Firstly, they dilate vessels to enhance blood flow. Secondly, they recruit pericytes to stabilise blood flow and maintain vessel oxygen partial pressure. Thirdly, they suppress HIF and VEGF expression, thereby inhibiting the process of angiogenesis and VM. Collectively, these actions increase intratumoural oxygen levels, leading to hypoxic tumour cell death and an improvement in oxygen utilisation efficiency. Created with BioRender.com.

PARP inhibitors can mitigate these adverse effects by inhibiting HIF activity [88] and reducing VEGF expression, thereby diminishing the formation of aberrant neovascularisation [89] and obstructing the development of VM [90]. These actions further reduce the oxygen supply of tumour cells, weaken their invasive ability, and ultimately lead to tumour cell necrosis. Additionally, PARP inhibitors contribute to vascular normalisation and increase pericyte coverage. Pericytes can enhance the endothelial barrier, prevent plasma extravasation, maintain vascular tension, prevent vascular collapse or overexpansion, stabilise blood flow, and enhance the oxygen partial pressure of vessels while reducing oxygen fluctuations [91] (Figure 5).

In summary, PARP inhibitors exhibit a dual mechanism of action: they reduce inefficient oxygen supply sources by inhibiting pathological angiogenesis and concurrently enhance oxygen delivery by reinforcing the homeostasis of existing functional vasculature. This dual effect ultimately elevates the oxygen level within the tumour microenvironment, thereby providing a foundation for enhancing radiotherapy sensitivity.

Moreover, PARP inhibitors have potential as carriers for radiopharmaceuticals, enabling targeted radiotherapy for tumours with high PARP expression. In in vitro studies, olaparib and lucaparib labelled with ^125^I demonstrated selective cytotoxicity against triple‐negative breast cancer and ovarian cancer cells, respectively [92, 93].

Other synthetic lethal drugs have also shown promise as radiosensitizers. Preclinical studies indicate that ATR inhibitors, including VE‐821 [94], berzosertib [95], and AZD6738 [96], may act as radiosensitizers in various cancer types. Specifically, VE‐821 has shown efficacy in pancreatic cancer, while berzosertib demonstrated radiosensitising effects in head and neck squamous cell carcinoma. These findings suggest that ATR inhibitors could provide an effective strategy for enhancing RT. Maximising the radiosensitising potential of synthetic lethal drugs remains a critical area of ongoing research.

## 5 The Combined Strategy of Synthetic Lethal Drugs and Immunotherapy

Traditional cancer treatments—chemotherapy, radiotherapy and surgery—remain fundamental, but tumour immunotherapy has emerged as a promising approach due to advancing understanding of tumour immune escape mechanisms. Tumour immunotherapy involves activating or enhancing the patient's immune system to recognise and attack tumour cells. This approach includes immune checkpoint inhibitors (ICIs), adoptive cellular immunotherapy and tumour vaccines.

Common immune checkpoints, such as programmed death‐1 (PD‐1)/programmed death‐ligand 1 (PD‐L1), cytotoxic T lymphocyte‐associated antigen‐4 (CTLA‐4) and lymphocyte activation gene‐3 (LAG‐3), are key molecules in the T‐cell co‐inhibitory signalling pathway. ICIs restore the cytotoxic activity of T cells by blocking these inhibitory signals [97]. ICIs have been approved for several cancer types, including melanoma [98], Hodgkin's lymphoma [99], non‐small cell lung cancer [100] and head and neck squamous cell carcinoma [97]. However, ICIs alone often yield limited efficacy, prompting researchers to explore strategies for combining immunotherapy with other targeted treatments.

PARP inhibitors have been reported to modulate the immunosuppressive microenvironment of tumours. By inhibiting cellular damage repair pathways, PARP inhibitors lead to the accumulation of DNA fragments, which subsequently activate the cyclic GMP‐AMP synthase‐stimulator of interferon genes (cGAS‐STING) pathway [101]. This activation drives the phosphorylation of transcription factors TBK1 and IRF3, regulating the transcription of cytokines such as interferons and chemokines (e.g., CCL5 and CXCL10). These cytokines act synergistically to recruit T‐cells, natural killer (NK) cells and antigen‐presenting cells (APCs), thereby enhancing the infiltration of tumour‐infiltrating lymphocytes (TILs) [102].

PARP inhibitors also upregulate PD‐L1 expression by inactivating GSK3β [103], regulating nuclear factor kappa‐B (NF‐κB) [104], and facilitating signal transducer and activator of transcription (STAT) signalling. Clinical studies have explored the potential of combining PARP inhibitors with immune checkpoint inhibitors. For instance, the MEDIOLA study [105], a phase II trial, evaluated the combination of the PARP inhibitor olaparib and the PD‐L1 inhibitor durvalumab in patients with platinum‐sensitive recurrent ovarian cancer. Similarly, the TOPACIO trial [106], another phase II study, investigated the effects of combining the PARP inhibitor niraparib with the PD‐1 inhibitor pembrolizumab in recurrent ovarian cancer. Both studies demonstrated the potential of such combinations as a treatment strategy.

Beyond PARP inhibitors, the ATR inhibitor ceralasertib, combined with the PD‐L1 antibody durvalumab, has shown significant clinical benefits in patients with melanoma and other cancers responsive to immunotherapy [107]. In a murine tumour model, intermittent treatment with ceralasertib induced upregulation of the type I interferon (IFN‐I) pathway, the primary mediator of its antitumour activity when combined with the PD‐L1 antibody.

However, despite these promising findings, challenges persist. For example, a phase II trial combining niraparib with the PD‐1 monoclonal antibody dostarlimab [108] reported limited efficacy and a deterioration in patients' quality of life with prolonged treatment. These findings underscore the complexities and obstacles in optimising combination regimens involving synthetic lethal drugs and immune checkpoint inhibitors.

## 6 The Combined Strategy of Synthetic Lethal Drugs and Hyperthermia

Hyperthermia (HT) is a therapeutic approach that utilises exogenous energy to elevate the temperature of specific body regions, thereby impairing the proliferative capacity of tumour cells. By heating tumour tissues to a therapeutic temperature and maintaining it for a specified duration, HT selectively induces apoptosis in tumour cells, which are less heat‐resistant than normal cells. This selective effect minimises damage to surrounding healthy tissues, making HT a promising cancer treatment modality. It has been increasingly applied in combination with chemotherapy and radiotherapy [109].

HT enhances tumour cell sensitivity to radiotherapy by influencing the cell cycle and improving the oxygenation of the tumour microenvironment [110]. Cells in the S phase, typically resistant to radiotherapy, are sensitive to HT, while hypoxic regions within tumours, which are also radioresistant, benefit from HT‐induced increased blood perfusion. This dual sensitising effect of HT and synthetic lethal drugs maximises the therapeutic outcomes of radiotherapy. Studies have demonstrated that combining radiotherapy, HT and PARP inhibitors in advanced cervical cancer cells significantly enhances anticancer efficacy while reducing side effects [80]. Furthermore, the combination of HT with PARP inhibitors [111] or WEE1 inhibitors [112] has shown therapeutic potential in ovarian cancer.

HT also inhibits BRCA2, a key protein in the homologous recombination (HR) repair pathway [113]. This suggests that, in the treatment of non‐BRCA1/2‐mutated tumours, HT can transiently suppress BRCA2 protein expression to mimic a BRCA‐deficient state. This functional feature of HT broadens the application of PARP inhibitors to BRCA wild‐type tumours, thereby extending treatment options to a wider range of cancer patients.

## 7 Conclusion

**FIGURE 6:**
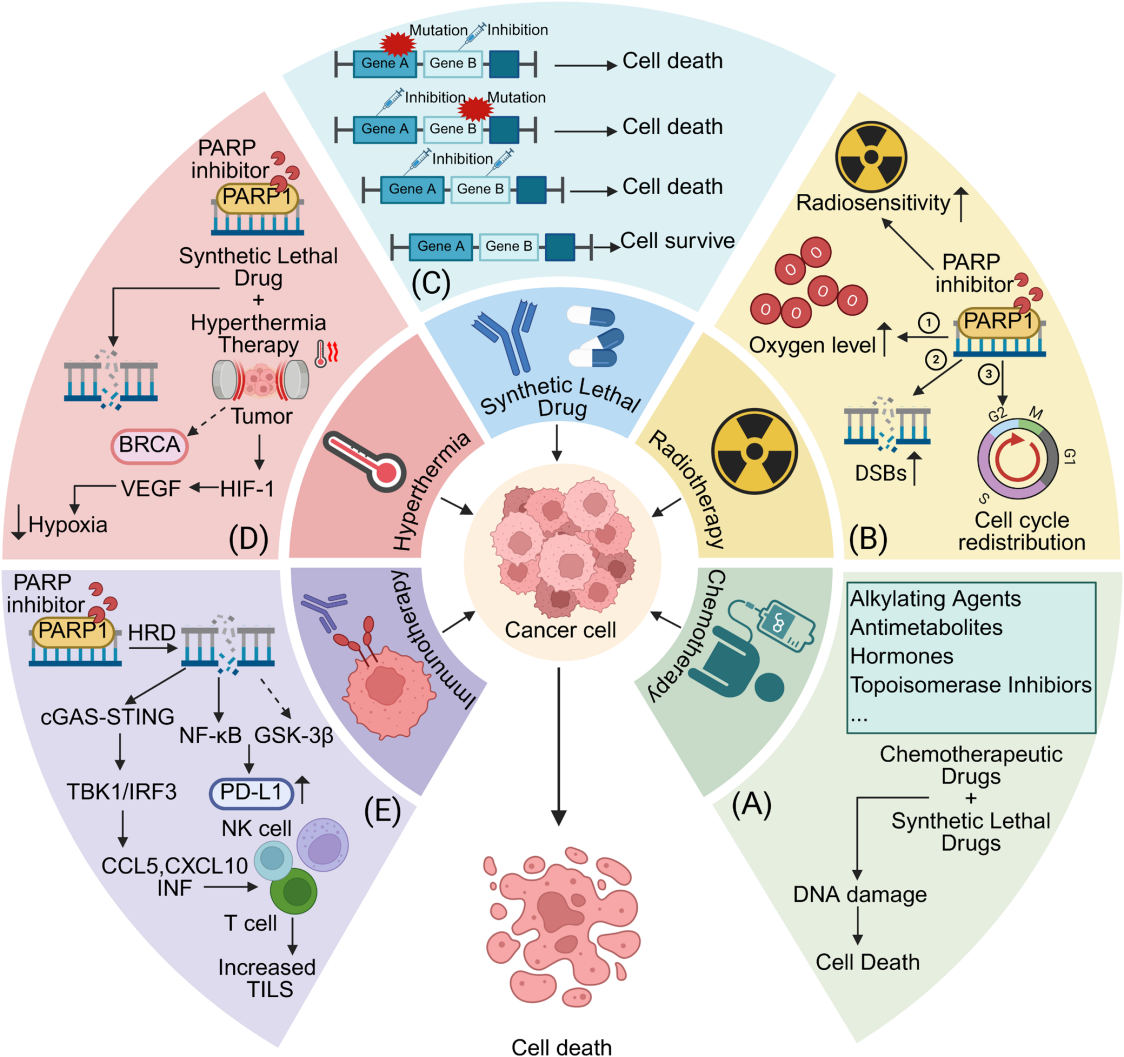
The combined strategy of synthetic lethal drugs with traditional cancer treatments. (A) The combination of synthetic lethal drugs and chemotherapeutic drugs enhances the damage to tumour cells. (B) Synthetic lethal drugs can enhance the sensitivity of cells to radiotherapy by increasing microenvironmental oxygen levels, distributing the cell cycle, and accumulating double‐strand breaks (DSBs). (C) The principle of synthetic lethality. (D) The combination of synthetic lethal drugs and hyperthermia can improve cellular hypoxia and broaden the spectrum of applications for PARP inhibitors. (E) PARP inhibitors can increase tumour‐infiltrating lymphocytes and up‐regulate the expression of PD‐L1, which improves the efficacy of immunotherapy. Created with BioRender.com.

The evolution of cancer treatment from conventional modalities to precision therapies has introduced synthetic lethal drugs as a more targeted and individualised approach. These drugs, when combined with traditional treatments, address issues of patient tolerance to conventional therapies while expanding the scope of application (Figure 6). Advances in drug screening technologies, such as yeast screening, RNA interference (RNAi), CRISPR‐Cas9 and bioinformatics‐based identification methods, have provided robust platforms for identifying stable and reliable synthetic lethal gene pairs. With the increasing understanding of the DNA damage response (DDR) pathway, new synthetic lethal gene pairs have emerged and demonstrated promising clinical utility. Additionally, synthetic lethal interactions outside the DDR pathway, such as PRMT5 inhibitors and MAT2A inhibitors, offer new directions for precision therapies.

While synthetic lethal drugs, particularly PARP inhibitors, have significantly impacted clinical treatment, challenges such as drug resistance and adverse events remain critical issues. Efforts to overcome these challenges by improving drug resistance and ensuring safety and efficacy are essential for advancing their clinical application.

The concept of synthetic lethality continues to evolve. Researchers have identified that cellular and organismal phenotypes are influenced by polygenic effects and complex interactions, suggesting that synthetic lethality extends beyond interactions between two genes. This has led to the development of the concept of complex synthetic lethality [114], which considers multifactorial genetic interactions and environmental vulnerabilities in tumour cells. This expanded framework not only enhances our understanding of gene collaboration in regulating cellular phenotypes but also provides a foundation for developing safer and more effective anticancer therapies.

## Author Contributions

**Lingya Wu:** writing – original draft (lead). **Yixuan Deng:** visualization (supporting). **Zhe Lei:** writing – review and editing (equal). **Yuhong Wang:** supervision (equal). **Shan Huang:** supervision (equal).

## Conflicts of Interest

The authors declare no conflicts of interest.

## Data Availability

The authors have nothing to report.
